# Modeling risks of cardiovascular and cancer mortality following a diagnosis of loco-regional breast cancer

**DOI:** 10.1186/s13058-021-01469-w

**Published:** 2021-09-27

**Authors:** Nicole M. Leoce, Zhezhen Jin, Rebecca D. Kehm, Janise M. Roh, Cecile A. Laurent, Lawrence H. Kushi, Mary Beth Terry

**Affiliations:** 1 Department of Epidemiology, Joseph L. Mailman School of Public Health, Columbia University, 722 West 168th Street, New York, NY 161110032 USA; 2 Division of Research, Kaiser Permanente Northern California, Oakland, CA USA

**Keywords:** Breast cancer, Cardiovascular disease, Cardiovascular mortality, Risk prediction models

## Abstract

**Background:**

Many women with breast cancer also have a high likelihood of cardiovascular mortality, and while there are several cardiovascular risk prediction models, none have been validated in a cohort of breast cancer patients. We first compared the performance of commonly-used cardiovascular models, and then derived a new model where breast cancer and cardiovascular mortality were modeled simultaneously, to account for the competing risk endpoints and commonality of risk factors between the two events.

**Methods:**

We included 20,462 women diagnosed with stage I–III breast cancer between 2000 and 2010 in Kaiser Permanente Northern California (KPNC) with follow-up through April 30, 2015, and examined the performance of the Framingham, CORE and SCOREOP cardiovascular risk models by area under the receiver operating characteristic curve (AUC), and observed-to -expected (*O*/*E*) ratio. We developed a multi-state model based on cause-specific hazards (CSH) to jointly model the causes of mortality.

**Results:**

The extended models including breast cancer characteristics (grade, tumor size, nodal involvement) with CVD risk factors had better discrimination at 5-years with AUCs of 0.85 (95% CI 0.83, 0.86) for cardiovascular death and 0.80 (95% CI 0.78, 0.87) for breast cancer death compared with the existing cardiovascular models evaluated at 5 years AUCs ranging 0.71–0.78. Five-year calibration for breast and cardiovascular mortality from our multi-state model was also excellent (*O*/*E* = 1.01, 95% CI 0.91–1.11).

**Conclusion:**

A model incorporating cardiovascular risk factors, breast cancer characteristics, and competing events, outperformed traditional models of cardiovascular disease by simultaneously estimating cancer and cardiovascular mortality risks.

**Supplementary Information:**

The online version contains supplementary material available at 10.1186/s13058-021-01469-w.

## Background

There are an estimated 3.8 million breast cancer (BC) survivors in the United States [1]. The majority of BCs are diagnosed as local or regional cancers (Stages I- III) and, given improvements in cancer screening and treatment, the 5-year survival rate for women with BC is now over 90% [1]. Many women diagnosed with BC will die from other causes, with cardiovascular disease (CVD) being the most common non-BC-related cause of death [2]. The probability of dying from a non-BC-related cause increases with age at BC diagnosis and in those with early stage BC [3]. BC survivors are at increased risk of CVD due to shared risk factors between cancer and CVD, as well as the cardiotoxicity of many cancer therapies [4]. In order to reduce health disparities in cardio-oncology outcomes, it is essential that we have a better way of risk stratifying women based on their CVD risk and BC treatment history. It is likely that the risk of cardiovascular disease and mortality [4, 5], as well as the risk of treatment related cardio-toxic effects in BC survivors is as a result underestimated by providers and women [4, 6, 7].

Given the expected long-term survival and increase in risk of cardiovascular events and mortality in BC survivors, prospective evaluation of the performance of risk models will help in clinical risk counseling by primary care physicians and oncologists. Currently, there are a number of CVD risk prediction models that have been developed for the general population [8–12]. However, some have been found to have poor performance in persons older than age 65 years, and especially in women [13–15]. The performance of these commonly-used CVD models in women with a history of BC has not been evaluated. To address this gap, we examined the performance of established CVD risk models in a non-metastatic BC population. We then developed an enhanced model to more accurately identify combinations of risk factors that may predict an increased risk for CVD versus BC mortality.

## Methods

### Study population and data collection

Our study population included all women who were diagnosed for the first time with stages I to III BC in the Kaiser Permanente Northern California (KPNC) healthcare delivery system from January 1, 2000 to December 31, 2010, with follow-up through April 30, 2015. The KPNC coverage area includes 23 counties in the San Francisco Bay Area and the Central Valley of California [16, 17]. We linked data from the KPNC Cancer Registry to the electronic medical record (EMR) and administrative and clinical electronic databases within KPNC to obtain patient demographic, cancer specific, and model covariate information. This study including the analytic data plan was approved by the KPNC Institutional Review Board.

Using the medical record, we defined patient age as age at first diagnosis of BC, race as White, Black, Asian/Pacific Islander, or Other, and ethnicity as Hispanic or non-Hispanic. Information on BC included type of surgery (lumpectomy, mastectomy (unilateral or bilateral)), stage, grade, lymph nodes (number examined and number positive), estrogen receptor (ER) and progesterone receptor (PR) status, HER2 status, tumor size (cm), and laterality. Information on treatment was also extracted from the medical record, including chemotherapy (within the first year of diagnosis) and hormonal therapy. Treatment variables included whether or not the patient received any radiation, chemotherapy, or hormonal therapy. For chemotherapy we also extracted intravenous medications received, and summarized by drug class. Using data from the medical record, we summarized the patients’ other comorbidities based on the modified Charlson Comorbidity Index [18].

We extracted the first recorded measurement of CVD risk factors for this patient population in the time frame of 6 months prior to cancer diagnosis to up to 18 months post diagnosis. We extracted the following information needed for the CVD models: total cholesterol, HDL cholesterol, LDL cholesterol, systolic blood pressure (average of 2 measurements if available), smoking status, diabetes, and whether prescribed any blood pressure lowering medication. We also recorded history of CVD defined as the occurrence of any of the following ICD-9 codes at any time prior to, up through 6 months post, BC diagnosis [19] (ICD-9 codes: 410–414, 428, 431, 432, 434, 435, 440.21). We include both CVD mortality and morbidity.

We created person-time data based on date of diagnosis of BC until either disenrollment from KPNC (defined as > 90-day lapse in enrollment), date and cause of death (available in the KPNC Cancer Registry), or end of follow-up (April 30, 2015), whichever came first. Cardiovascular death also included ICD-10 codes: I00-I99.

### CVD model selection

Models were first selected based on a systematic review of the literature [20] and were limited to those that could be validated based on availability of data from the KPNC EMR. A summary of included models can be found in Additional file 1: Table S1. The majority of the models were developed on the Framingham cohort and predicted the endpoint “hard CHD events", defined as coronary death or CHD events (ICD-9 codes 402, 410–414, 429.2, and 429.9, see Additional file: Table S2 for full details). Additional focus was given to any models developed specifically for an older population (age > 65 years), which included the Systematic Coronary Risk Evaluation (SCORE) [8, 13] and Coronary Risk in the Elderly (CORE) [14]. Other CVD risk models that could not be validated due to their use of covariates that are not routinely captured in clinical practice included the Reynolds Risk Score, QRISK, PROCAM, and Framingham with additional covariates such as BMI and heart rate [21–26].

## Statistical methods

We first compared patient and disease characteristics using descriptive statistics and calculated and plotted cumulative incidences of death by cause. In order to assess the performance of the CVD models, individual patient risk scores were calculated based on the original models either by use of score sheets [19] or direct calculation from the model’s parameter estimates. We used Cox proportional hazards models to create the Framingham recalibrated model, keeping the established risk factor categories from the 2008 Framingham Model [9] and re-calculating the parameter estimates based on the current cohort.

We summarized discrimination of each model at the model’s recommended prediction interval by the area under the ROC curve (AUC), with 95% confidence intervals calculated using the methods of DeLong [27]. Due to the variation in endpoint definition (e.g. morbidity and mortality endpoints) and prediction fixed-time horizons (e.g. 5 year versus 10 year) across models, we used two approaches to calculate the area under the ROC curve (AUC) for each model. The first approach we used was a naïve approach in which we excluded patients who were not followed for the full prediction horizon either due to experiencing a competing cause of death or shorter follow-up time. In this approach, patients who experienced an event after the prediction time frame were considered non-events. The second approach we applied used all patients, and those who did not have complete follow-up for the prediction horizon contributed data only up to the length of time that they were followed, such that competing events are not excluded from the validation sample, but rather censored at their last follow-up time [28]. We used the *rmap* package in R for this latter approach. For model validation analyses, if a patient was missing any covariate data necessary for the risk calculation, they were excluded from the validation of that specific model.

In creating a new model, we used a multi-state framework that allows for the simultaneous modeling of the competing causes of death from BC, CVD, or all other causes. Each cause of death is considered a state to which the patient can transition after diagnosis, with a given probability based on their individual risk profile. The transitions are modeled with stratified (or *cause-specific*) Cox proportional hazards models, using the *mstate* library in R [29–32]. We considered risk factors from the established CVD models as well as BC characteristics, treatment indicators, age, and history of CVD before BC. In order to include the greatest number of women in our new model, we allowed a “missing” category to be included for each covariate that contained missing EMR data. We included in the final models only risk factors that were statistically significantly associated with the transition of interest. As sensitivity analyses we also created separate Cox proportional hazards models and sub-distribution hazards models [33] for CVD and BC death. Lastly, to examine the impact of missing risk factor data, complete case analyses were run, which included only those observations with non-missing values for all risk factors in the models. We summarized model discrimination by averaging the AUC from 10 bootstrap samples of our original data. We used SAS version 9.4 for Windows (SAS Institute, Cary, NC) and R version 3.1.2 (http://cran.us.r-project.org/) for all analyses.

## Results

There were 20,462 women diagnosed with stage I-III BC between 2000 and 2010 in KPNC (Table 1). The mean age at diagnosis was 60 years (range 21 to 103 years). The majority of the sample was white (79%) and non-Hispanic (90%). Over half the sample (52%) was diagnosed with stage 1 disease, and nearly all were treated with surgery (57% lumpectomy, 41% mastectomy).

**Table 1:** Patient baseline and disease characteristics of Kaiser Permanente Northern California Cohort (KPNC) Stage I–III breast cancers, diagnosed 2000–2010

	All patients (*N* = 20,462) *N* (%)	Breast Cancer Death (*N* = 842)	CVD Death (*N* = 696)
**Age** (years)			
21–34	302 (1.5%)	13 (1.5%)	3 (0.4%)
35–39	571 (2.8%)	36 (4.3%)	8 (1.2%)
40–44	1340 (6.6%)	65 (7.7%)	12 (1.7%)
45–49	2167 (10.6%)	81 (9.6%)	21 (3.0%)
50–54	2604 (12.7%)	90 (10.7%)	33 (4.7%)
55–59	2962 (14.5%)	104 (12.4%)	39 (5.6%)
60–64	2866 (14.0%)	106 (12.6%)	52 (7.5%)
65–69	2482 (12.1%)	78 (9.3%)	71 (10.2%)
70–74	2004 (9.8%)	81 (9.6%)	109 (15.7%)
75 +	3164 (15.5%)	188 (22.3%)	348 (50.0%)
**Race**			
Non-Hispanic White	14,181 (69.3%)	601 (71.4%)	531 (76.3%)
Hispanic White	1985 (9.7%)	77 (9.1%)	38 (5.5%)
Non-Hispanic Black	1500 (7.3%)	81(9.6%)	85 (12.2%)
Hispanic Black	7 (< 0.1%)	0	0
Asian/Pacific Islander	2719 (13.3%)	80 (9.5%)	40 (5.8%)
Other/Unknown	70 (0.3%)	3 (0.4%)	2 (0.2%)
**Smoking status at diagnosis**			
Current smoker	2430 (11.9%)	109 (13.0%)	126 (18.1%)
Former smoker	3095 (24.0%)	100 (11.9%)	97 (13.9%)
Non-smoker	7389 (36.1%)	162 (19.2%)	97 (13.9%)
Unknown	7548 (36.9%)	471 (55.9%)	376 (54.0%)
**Diabetes**	2669 (13.1%)	104 (12.4%)	160 (23.0%)
**Charlson comorbidity**			
0	13,130 (64.2%)	544 (64.6%)	277 (39.8%)
1–2	6137 (30.0%)	240 (28.5%)	288 (41.4%)
3 +	1163 (5.7%)	56 (6.7%)	130 (18.7%)
unknown	32 (0.2%)	2 (0.2%)	1 (0.1%)
**Breast Cancer Characteristics**			
**Bilateral** **Unilateral—Right** **Unilateral-Left** **Unilateral—Unknown**	138 (0.7%) 9842 (48.1%) 10,476 (51.2%) 6 (< .01%)	4 (0.5%)	8 (1.2%)
**Surgery**			
None	560 (2.7%)	59 (7.0%)	39 (5.6%)
Lumpectomy	11,583 (56.6%)	270 (32.1%)	321 (46.1%)
Mastectomy	8309 (40.6%)	512 (60.8%)	334 (48.0%)
Unknown	10 (0.1%)	1 (0.1%)	2 (0.3%)
**Grade**			
1 Well differentiated	4734 (23.1%)	37 (4.4%)	130 (18.7%)
2 Moderately differentiated	8336 (40.7%)	287 (34.1%)	272 (39.1%)
3 Poorly differentiated	5481(26.8%)	424 (50.4%)	198 (28.5%)
4 Diffuse	259 (1.3%)	20 (2.4%)	7 (1.0%)
Unknown	1652 (8.1%)	74 (8.8%)	89 (12.8%)
**Stage**			
1	10,843 (53.0%)	127 (15.1%)	294 (42.3%)
2	7806 (38.2%)	444 (52.7%)	316 (45.4%)
3	1813 (8.9%)	271 (32.2%)	86 (12.4%)
**Tumor size**			
≤ 2 cm	13,727 (67.1%)	269 (32.0%)	393 (56.5%)
(2,5] cm	5723 (28.0%)	411 (48.8%)	244 (35.1%)
> 5 cm	838 (4.1%)	117 (13.9%)	44 (6.3%)
Diffuse or Unknown	174 (0.9%)	45 (5.3%)	15 (2.2%)
**ER/PR**			
Positive	11,917 (58.2%)	393 (46.7%)	442 (63.5%)
Negative	2689 (13.1%)	253 (30.1%)	105 (15.1%)
Unknown/not done	5856 (28.6%)	196 (23.3%)	149 (21.4%)
**HER2**			
Positive	1862 (9.1%)	110 (13.1%)	49 (7.0%)
Negative	11,938 (58.3%)	422 (50.1%)	417 (59.9%)
Unknown/not done	6662 (32.6%)	310 (36.8%)	230 (33.1%)
**Positive Lymph Nodes**			
0	14,150 (69.2%)	327 (71.1%)	449 (64.5%)
1–3	4429 (21.7%)	234 (21.3%)	156 (22.4%)
> 3	1851 (9.1%)	279 (7.5%)	87 (12.5%)
None examined/unknown	32 (0.2%)	2 (0.1%)	4 (0.6%)
**Treatments received**			
Any chemotherapy	9639 (47.1%)	596 (70.8%)	204 (29.3%)
Taxanes	2806 (13.7%)	220 (26.1%)	47 (6.8%)
Targeted Therapy	1206 (5.9%)	78 (9.3%)	16 (2.3%)
Alkylating Agents	1626 (8.0%)	113 (13.4%)	41 (5.9%)
Antimetabolites	1061 (5.2%)	174 (20.7%)	33 (4.7%)
Vinca Alkaloids	364 (1.8%)	94 (11.2%)	14 (2.0%)
Other	1174 (5.7%)	133 (15.8%)	23 (3.3%)
No Chemotherapy	10,823 (52.9%)	246 (29.2%)	492 (70.7%)
Any hormonal therapy	8463 (41.4%)	265 (31.5%)	282 (40.5%)
Any radiation	6621(32.4%)	247(29.3%)	184 (26.4%)

There were 17,773 (86.9%) women who were alive at last follow-up (in 2015 or at the time of censoring). Less than 1% were censored for disenrollment from KPNC, and the median follow-up for survivors was 7.5 years. There were 2729 (13.3%) deaths overall, with 842 (4.1% of the cohort or 38.5% of deaths) due to BC and 696 (3.4% of the cohort or 25.5% of deaths) due to cardiovascular events. Of the 1191 (5.8% of the cohort or 43.6% of deaths) deaths from other causes, the largest subgroups were due to other cancers (*n* = 321) and unspecified respiratory causes (*n* = 198). (See Additional file: Table S3 for a summary of the non-fatal cardiovascular events following a BC diagnosis.)

**Fig. 1:**
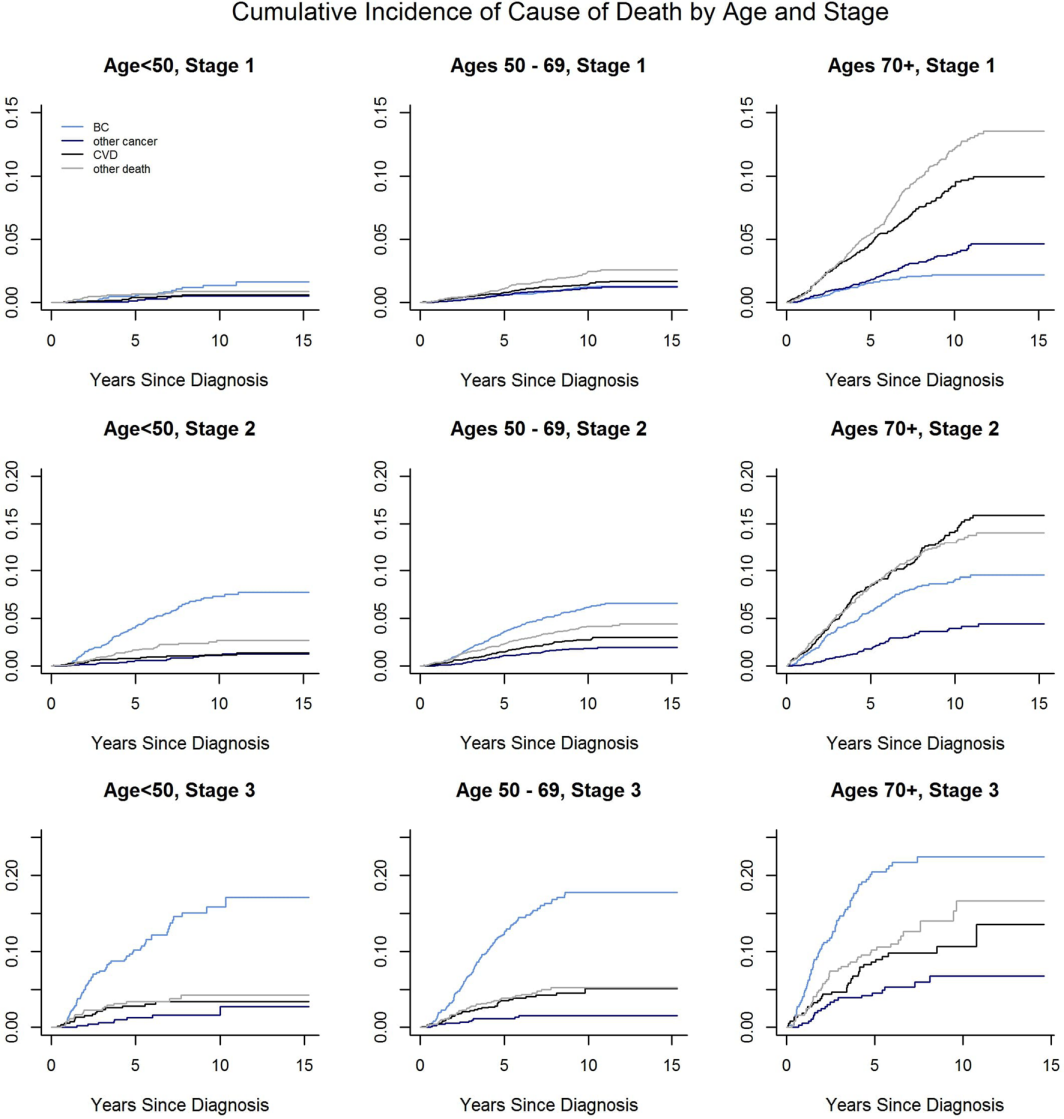
Cumulative incidence of cause of death, by stage and age at breast cancer diagnosis

In this cohort, there were also differences in cause of mortality by age and stage at BC diagnosis. For those less than 50 years of age at BC diagnosis, and those with stage 3 disease, BC was the leading cause of death (see Fig. 1). For those greater than 70 years at diagnosis with stage 1 and 2 disease, the cumulative incidences of mortality from CVD and all other causes were much higher than those due to BC. Women who were diagnosed in the 50–69 age range also had survival outcomes that were dependent upon their stage at BC diagnosis, with stage 2 and 3 disease having higher incidences of BC death.

Table 2 summarizes the discrimination of existing CVD models whose AUCs ranged from 0.64 (Framingham model for hard events evaluated at 10 years) to 0.78 (Framingham model recalibrated to our data set, evaluated at 5 years) in the current cohort. The SCORE OP model, developed specifically for use on an older population (age > 65), did not outperform the models developed on a broader range of age groups (SCORE OP AUC = 0.76, 95% CI 0.73–0.81), nor did the CORE model which was the only model accounting for competing causes of death (AUC = 0.74, 95% CI 0.72, 0.76).

**Table 2:** Performance of CVD risk models by end-point and statistical method, KPNC Stage I-III breast cancers, diagnosed 2000–2010

Risk Model (model type)	Evaluation time-point (years)	Events	Validation Sample Size* (naïve)	AUC (95% CI) (naïve)	AUC (95% CI) (with censoring)	O/EO ratio (95% CI)
**Outcome: Hard CHD (fatal and non-fatal: Myocardial Infarction (MI), coronary insufficiency)**						
Framingham 2000 (Weibull AFT model)	2	304	10,211	0.70 (0.67, 0.73)	0.63 (0.61, 0.66)	0.92 (0.83, 1.03)
	4	596	9529	0.74 (0.72, 0.76)	0.74 (0.72, 0.76)	0.85 (0.78, 0.92)
Framingham 2001 (Proportional Hazards (PH)Model)	5	699	8236	0.71 (0.69, 0.73)	0.70 (0.69, 0.73)	1.98 (1.83, 2.12)
	10	952	1976	0.64 (0.62, 0.67)	0.65 (0.62, 0.67)	1.06 (0.99, 1.13)
Framingham recalibrated (PH model)	5	699	8236	0.78 (0.76, 0.80)	0.77 (0.75, 0.79)	0.31 (0.29, 0.34)
	10	952	1976	0.76 (0.74, 0.79)	0.74 (0.72, 0.76)	0.29 (0.27, 0.31)
CORE (sub-distribution hazards model)	5	692	8180	0.75 (0.73, 0.77)	0.74 (0.72, 0.76)	3.30 (3.07, 3.56)
	10	943	1963	0.75 (0.73, 0.78)	0.73 (0.71, 0.76)	1.72 (1.61, 1.83)
**Outcome: Earliest of Any** **(MI, coronary death, coronary insufficiency, angina, stroke, peripheral artery disease)**						
Framingham 2008 (PH Model)	10	3734	4478	0.66 (0.64, 0.68)	0.71 (0.70, 0.73)	3.53 (3.42, 3.64)
**Outcome: CVD death only**						
SCORE (Weibull AFT model)	10	184	1300	0.73 (0.69, 0.78)	0.74 (0.70, 0.78)	0.44 (0.38, 0.51)
SCORE OP (PH model)	10	184	1300	0.76 (0.72, 0.80)	0.76 (0.73, 0.81)	0.22 (0.19, 0.25)

^*^denotes complete risk factor information and required follow-up time for model evaluation

Despite the moderate discriminative ability of the existing models, the O/E ratios show that the models for 5 and 10-year predictions, those predicting fatal events only, and those developed on a European population were not well calibrated to this cohort of BC patients. The majority of the models tended to underestimate the number of events across most deciles of risk, while the recalibrated models over-estimated the number of events. Those with shorter prediction horizons (2–4 years) had better calibration, and may therefore be more useful in this patient population.

## Multi-state model

To evaluate whether we could improve upon the previously published CVD models for a cohort of early-stage BC women, we fit a multi-state model including both established cardiovascular risk factors, and BC disease and treatment characteristics (see Table 3). The corresponding hazard ratios appear in Additional file 1: Table S7. BC characteristics including grade, ER/PR status, tumor size, and nodal involvement were all significantly associated with risk of BC death (*p* < 0.05), as expected, but also associated with CVD and other-cause mortality as well. Older age, current and former smoking, HDL cholesterol < 35 mg/dl, ER negative disease, and having no surgery for BC were also associated with an increased risk of each cause of death. Prior history of CVD, higher Charlson comorbidity score, and lack of receipt of radiation or chemotherapy were associated with an increased risk of death from CVD and death from other causes, but not death due to BC. Due to small numbers of events within strata, and multivariate endpoint, we were unable to test individual drug classes in this model. In sensitivity analyses, the results from separate Cox models and sub-distribution hazards models for each cause of death, yielded similar results to the multi-state model. The complete case analyses also provided similar inferences, though with decreased statistical power (results not shown).

**Table 3:** Multi-state Model for Cause of Death, KPNC Cohort Stage I-III breast cancers, diagnosed 2000–2010

	CVD Mortality 696 events HR (95%CI)	Breast Cancer Mortality 842 events HR (95% CI)	All Other Causes 1191 events HR (95%CI)
**Age at diagnosis categories**			
21–39	Reference	Reference	Reference
40–49	0.81 (0.13,1.49)	1.09 (0.76, 1.42)	0.63 (0.21, 1.05)
50–54	1.03 (0.34,1.72)	0.97 (0.62, 1.32)	0.65 (0.21, 1.09)
55–59	1.04 (0.36,1.72)	1.11 (0.77, 1.45)	1.02 (0.61, 1.43)
60–64	1.34 (0.68,2.00)	1.30 (0.96, 1.64)	1.04 (0.63, 1.45)
65–69	1.88 (1.23,2.53)	1.19 (0.83, 1.55)	1.44 (1.04, 1.84)
70–74	3.40 (2.76,4.04)	1.65 (1.29, 2.01)	2.45 (2.06, 2.84)
75 +	6.27 (5.64,6.90)	2.47 (2.15, 2.79)	4.02 (3.63, 4.41)
**Race**			
White	Reference	Reference	Reference
Black	1.78 (1.55, 2.01)	1.27 (1.04, 1.50)	1.13 (0.92,1.34)
Asian/Pacific Islander	0.70 (0.37, 1.03)	0.77 (0.53, 1.01)	0.68 (0.44, 0.92)
Other/Unknown	1.04 (0.35, 2.43)	1.27 (0.13, 2.41)	0.96 (0.18, 2.10)
**Smoking Status**			
Current	2.59 (2.32,2.86)	1.96 (1.71, 2.21)	3.08 (2.87, 3.29)
Former	1.36 (1.09,1.63)	1.33 (1.08, 1.58)	1.49 (1.28, 1.70)
Non-smoker	Reference	Reference	Reference
Unknown	2.28 (2.07,2.49)	2.29 (2.11, 2.47)	2.69 (2.52, 2.86)
**History of CVD**			
No prior history	Reference	–	Reference
History of event	2.10 (1.89,2.31)	–	1.28 (1.11, 1.45)
Unknown	1.53 (1.19,1.87)	–	1.44 (1.17, 1.71)
**HDL Cholesterol**			
< 35	1.54 (1.14,1.94)	1.92 (1.59, 2.25)	1.91 (1.62, 2.20)
35–44	1.04 (0.79,1.29)	1.28 (1.07, 1.49)	1.14 (0.95, 1.33)
45–49	0.84 (0.54,1.14)	0.90 (0.65, 1.15)	1.04 (0.83, 1.25)
50–59	0.81 (0.58,1.04)	0.99 (0.80, 1.18)	0.97 (0.80, 1.14)
60 +	Reference	Reference	Reference
Unknown	1.32 (1.06, 1.58)	1.57 (1.36, 1.78)	1.49 (1.27, 1.71)
**Charlson comorbidity**			
0	Reference	–	Reference
1–2	1.44 (1.26,1.62)	–	1.54 (1.41, 1.67)
3 +	2.28 (2.03,2.53)	–	2.55 (2.34, 2.76)
**Grade**			
Well differentiated	Reference	Reference	Reference
Moderately differentiated	1.11 (0.90,1.32)	2.86 (2.51, 3.21)	1.18 (1.02, 1.34)
Poorly differentiated	1.45 (1.20,1.70)	4.34 (3.99, 4.69)	1.56 (1.37, 1.75)
Diffuse	1.05 (0.28,1.82)	4.59 (4.03, 5.15)	1.02 (0.43, 1.61)
Unknown	1.52 (1.24, 1.80)	2.63 (2.23, 3.03)	1.12 (0.89, 1.35)
**Tumor size**			
≤ 2 cm	Reference	Reference	Reference
(2,5] cm	1.43 (1.25,1.61)	2.13 (1.96, 2.30)	1.38 (1.24, 1.52)
> 5 cm	1.91 (1.57, 2.25)	3.19 (2.95, 3.43)	2.01 (1.75, 2.27)
Diffuse or Unknown	3.74 (3.20,4.28)	5.26 (4.92, 5.60)	2.91 (2.43, 3.39)
**Lymph Nodes Involved**			
0	Reference	Reference	Reference
1–3	1.23 (1.03,1.43)	1.71 (1.54, 1.88)	1.13 (0.98, 1.28)
4–9	1.51 (1.21,1.81)	3.04 (2.83, 3.25)	1.55 (1.32, 1.78)
10 +	2.35 (1.95,2.75)	6.48 (6.25, 6.71)	1.90 (1.58, 2.22)
Unknown	2.52 (1.51,3.53)	2.13 (0.72, 3.54)	–
**Radiation**	0.82 (0.64,0.99)	–	0.87 (0.73, 1.01)
**Chemotherapy**	0.77 (0.55,0.99)	–	0.83 (0.66, 1.00)
**Surgery**			
None	1.95 (1.59,2.31)	3.25 (2.95, 3.55)	1.98 (1.69, 2.27)
Lumpectomy	Reference	Reference	Reference
Mastectomy	1.05 (0.88,1.22)	1.33 (1.17, 1.49)	1.07 (0.94, 1.20)
Unknown	3.35 (1.93,4.77)	1.96 (0.03, 3.95)	–
**ER/PR**			
Positive	0.82 (0.59,1.05)	0.48 (0.31, 0.65)	0.77 (0.60, 0.94)
Negative	Reference	Reference	Reference
Unknown/not done	0.67 (0.41,0.93)	0.53 (0.34, 0.72)	0.63 (0.43, 0.83)

To evaluate the performance of the model using the AUC, predicted probabilities for the outcomes of interest were calculated at 3, 5 and 10 years for all patients in the data set (Additional file 1: Table S4). Similar to the established CVD risk scores, the model performs slightly better for the shorter time frame, with AUCs of 0.84 and 0.82 at 3 years versus 0.82 and 0.77 at 10 years, for CVD mortality and BC mortality, respectively, though performance of the new model remains higher overall. The observed to expected number of combined BC and CVD deaths at 5 years predicted by the multi-state model was also quite accurate (*O*/*E* = 1.01, 95% CI 0.91–1.11).

## Discussion

In this cohort of women diagnosed with breast cancer, the death rates from cardiovascular events and BC were roughly equivalent overall, though younger women were more likely to die of BC than cardiovascular events. Although our modeling was limited to a single health care organization, the KPNC cohort was very similar in age and other demographics to the U.S. SEER18 data (see Additional file: Table S8), with few exceptions (e.g., SEER18 has a higher percentage of Stage 3 (13.7 vs. 8.9) and women 75 + (19.8 vs. 15.5 compared to the KPNC cohort)).

Determining which BC survivors are at the greatest risk for CVD is of paramount importance in treating the entire patient, following her BC diagnosis, particularly given the shared risk factors for BC and CVD coupled with the cardiotoxic effects of many cancer therapies [4]. Yet, commonly used models for predicting CVD have not been prospectively validated in BC patients. Some important limitations of such models are that they were developed on cohorts that were younger and without a history of CVD events or comorbidities. The fact that none have been validated in a BC population is likely due in many settings to the lack of data on CVD risk factors such as blood pressure and lipids, collected around the time of diagnosis. To our knowledge, our study is the first to attempt to validate the use of these models following a loco-regional BC diagnosis. A key conclusion of our study is that an integrated model with both breast cancer characteristics and CVD risk factors improves prediction of CVD after a diagnosis of breast cancer.

We demonstrated that standard CVD prediction models showed moderate discrimination in our cohort, though performance was widely influenced by the time horizon for prediction of CVD outcome with better performance in shorter time horizons, likely due to the smaller risk of a competing death in that time frame and more complete follow-up of patients. The models performed better using a shorter time window and with more recent risk factor information suggesting that they could be used clinically at regular windows while women are still undergoing BC treatment.

The most common BC molecular subtype, hormone receptor positive (HR +) cancers, are typically treated for 5–10 years with endocrine therapies. This means that women are seen regularly for breast cancer treatment and thus our models which performed best in shorter time horizons could be feasible as many women are still being actively followed and treated. Therefore, in evaluating these models, we conclude that they may be useful in most women, particularly after age 65 years when women affected with BC may be more likely to die of CVD than BC [34], and recommend that no more than a 2–4 year prediction window be calculated based on these models. The limited utility of standard CVD prediction models for long-term risk prediction in BC survivors is concerning, especially given that higher CVD risk in BC survivors compared with the general population does not manifest itself until about 7 years after the diagnosis of BC [7].

Changes in definition of the outcome measure (e.g. morbidity versus mortality) across CVD models also present challenges in making comparisons of model performance. While moderate discrimination was seen in both the models predicting mortality and those predicting non-fatal events, it is not clear which endpoint may be more meaningful clinically in a cancer survivor population. For model validation, a mortality endpoint is less subjective, while non-fatal events may not be captured routinely outside of a clinical trial setting. Additionally, in our population, at least 14% of women already have a documented CVD event prior to their BC diagnosis and are likely being treated or monitored for a cardiovascular condition, thus predicting additional non-fatal events may be of less concern.

Another factor contributing to the poor performance of the established cardiovascular models could be differences in risk factor distribution, including missing risk factor data, between the original cohorts in which the models were developed, and our older, contemporary, comorbid cohort, as seen in Additional file: Table S5. For example, in the original Framingham cohort, 62% of women were non- smokers, versus only 36% in our current cohort, with an additional 37% missing smoking status and excluded from the validation analysis. Similarly, blood pressure-lowering medication was not routinely used in the original Framingham cohort, versus 27% of the current cohort having a history of its use, and subsequently, a lower overall distribution of blood pressure compared to the original Framingham women. However, even with recalibration of the Framingham model to our data set (Additional file 1: Table S6), model performance was not improved, likely due to the handling (ignoring) of deaths in the absence of a CVD event. This becomes especially problematic for longer-term prediction horizons, such as 5–10 years.

For longer-term prediction it is important to account for competing causes of mortality, thus this question supports the use of a multi-state model that can simultaneously model the competing failure types. Our findings that increased stage of BC at diagnosis was also significantly associated with CVD and all-cause mortality indicate that beyond traditional CVD risk factors and increased age. Whether this association between stage and CVD mortality reflects poorer underlying health in general by factors not measured in these models, barriers to health care access and quality (even within a large health care organization), and/or treatment related factors is unclear. However, what this association does suggest though is that patients with more advanced disease at any age should be aware of their increased risk of mortality from other causes following their primary BC treatment. For women less than age 50 years at BC diagnosis, managing the risk of BC recurrence and mortality remains of primary concern, but those with increased cancer burden may also be at elevated risk of CVD mortality, despite their young age. This could be in part, due to the early and delayed cardio-toxicities of the cancer treatment [4]. For example, the risk of heart failure is shown to increase with increasing cumulative doses of anthracyclines, which have been commonly used to treat early-stage BC for decades (5% versus 48% risk at a dose of 400 mg/m^2^ versus 700 mg/m^2^, respectively) (4). Incorporating detailed information about the treatment of BC into CVD risk prediction models, including the type, cumulative dose, and duration of therapy, might considerably improve performance. However, more work is needed in this area, given that we were unable to formally examine the relationship between individual drugs and outcomes using the multi-state model.

## Conclusions

We found that many BC prognostic factors were significantly associated with both BC mortality and CVD mortality. However, our data were limited by the fact that many of the women had prior cardiovascular events, may not have had blood lipids measured within the desired time frame of the BC diagnosis, were subject to missing data, such as detailed smoking and chemotherapy information, and were lacking additional known risk factors including BMI, diet and alcohol use. While our multi-state model requires further validation from an external source, it showed good discrimination in our cohort and might serve as a first line for identifying subgroups of patients that may be at increased risk of cardiovascular events based on readily accessible covariates.

## Supplementary Information

**Additional file 1**. Supplementary Tables.

## Abbreviations

AUC: Area under the curve
BC: Breast cancer
BMI: Body mass index
CHD: Coronary heart disease
CI: Confidence interval
CORE: Coronary Risk in the Elderly
CVD: Cardiovascular disease
EMR: Electronic medical record
ER: Estrogen receptor
HDL: High-density lipoprotein
HR +: Hormone receptor positive
ICD-9: International Classification of Diseases, Version 9
KPNC: Kaiser Permanente Northern California
LDL: Low-density lipoprotein
O/E Ratio: The observed-to-expected ratio
PR: Progesterone receptor
ROC: Receiver operating characteristic
SCORE: Systematic Coronary Risk Evaluation
SCORE OP: Systematic Coronary Risk Evaluation for older populations

## Declarations

### Availability of data and materials

The data used in this paper were deidentified data from Kaiser Permanente data. For approval to use Kaiser Permanente data please contact Dr. Lawrence Kushi (larry.kushi@kp.org).
