# Supplementary material for: Modeling risks of cardiovascular and cancer mortality following a diagnosis of loco-regional breast cancer
Source: Breast Cancer Res. 2021 Sep 27;23:91. doi: 10.1186/s13058-021-01469-w (PMC8474887; doi:10.1186/s13058-021-01469-w)
Supplement: Supplementary file 1 — Additional file 1. Supplementary Tables. [file 13058_2021_1469_MOESM1_ESM.docx]

**Supplemental Materials**

*Modeling risks of cardiovascular and cancer mortality following a diagnosis of loco-regional breast cancer*

Supplementary Table 1: Summary of included cardiovascular disease risk models

Supplementary Table 2: List of Cardiovascular Events and Associated ICD-9 Codes

Supplementary Table 3: Summary of outcomes and CVD events in the KPNC cohort.

Supplementary Table 4: AUCs of predicted probabilities calculated at 3, 5, and 10 years in the KPNC cohort

Supplementary Table 5: Cardiovascular disease risk factors in the KPNC cohort compared to Framingham women

Supplementary Table 6: Parameter estimates and relative risks for Framingham model recalibrated to KPNC data set on complete cases (N=11,019)

Supplementary Table 7: Hazard Ratios from the Multi-state Model of Cause of Death Following a Breast Cancer Diagnosis in KPNC Women

Supplementary Table. 8. Comparison of the KPNC to SEER18.

Supplementary Table 1: Summary of included cardiovascular disease risk models

| **Risk Model** | **Outcome definition** | **Years**  **predicted** | **Validation**  **Sample size*** | **Events** | **AUC**  **(95% CI)** |
| --- | --- | --- | --- | --- | --- |
| Framingham  2000 | Hard CHD | 2 (chart) | 10,211 | 304 | 0.70 (0.67, 0.73) |
|  | Hard CHD | 4 (Weibull) | 9,529 | 596 | 0.74 (0.72, 0.76) |
| Framingham  2001 | Hard CHD | 5 | 8,236 | 699 | 0.71 (0.69, 0.73) |
|  | Hard CHD | 10 | 1,976 | 952 | 0.64 (0.62, 0.67) |
| Framingham recalibrated | Hard CHD | Continuous | 11,019 | 966 | 0.62^¥^(0.56, 0.68) |
|  | Hard CHD | 5 | 8,236 | 699 | 0.78 (0.76, 0.80) |
|  | Hard CHD | 10 | 1,976 | 952 | 0.76 (0.74, 0.79) |
| Framingham  2008 | Earliest of  Any event | 10 | 4,478 | 3,734 | 0.66 (0.64, 0.68) |
| SCORE | CVD death | 10 | 1,300 | 184 | 0.73 (0.69, 0.78) |
| SCORE OP | CVD death | 10 | 1,300 | 184 | 0.76 (0.72, 0.80) |
| CORE | Hard CHD | 5 | 8,180 | 692 | 0.75 (0.73, 0.77) |
|  | Hard CHD | 10 | 1,963 | 943 | 0.75 (0.73, 0.78) |

*denotes complete risk factor information and required follow-up time

^¥^ Harrell C-Index

Supplementary Table 2: List of Cardiovascular Events and Associated ICD-9 Codes

| **Event Type** | **Associated ICD-9 Codes** |
| --- | --- |
| Myocardial Infarction | 410, 412 |
| Coronary insufficiency | 411, 414 |
| Angina pectoris | 413 |
| Ischemic stroke | 434 |
| Hemorrhagic stroke | 431, 432 |
| Transient ischemic attack | 435 |
| Congestive heart failure | 428 |
| Intermittent claudication | 440.21 |

Supplementary Table 3: Summary of outcomes and CVD events in the KPNC cohort.

| **Event type** | **N (%)** | **Median time to event**  **in months (IQR)** |
| --- | --- | --- |
| Disenroll from KP | 209 (1.0%) | 28 (15 – 50) |
| Deaths | 2,729 (13.3%) | 44 (23-69) |
| **Cause of death:** |  |  |
| Breast cancer | 842 (5.3%) | 40 (38 - 43) |
| Other cancer | 321 (2.3%) | 51 (47 - 55) |
| Heart disease/failure/stroke | 696 (4.7%) | 43 (40 – 46) |
| All other causes | 870 (5.9%) | 45 (42 – 48) |
|  |  |  |
| **Survivors** | 17,733 (81.8%) | 7.5 years (5.2 – 10.5) |
| >5 years follow-up | 13,685 (66.9%) | - |
| >10 years follow-up | 5,106 (25.0%) | - |
| **Summary of non-fatal CVD events** | | |
| Any Non-fatal CVD event | 8,023 (74.2%) | 52 (22 – 90) |
| CHD event | 2,395 (22.5%) | 35 (15 – 68) |
| Stroke | 2,637 (30.9%) | 56 (28 – 91) |
| Peripheral Artery Disease | 5,738 (71.3%) | 79 (49 – 117) |
| Heart Failure | 2,277 (23.2%) | 40 (16 – 76) |

Supplementary Table 4: AUCs of predicted probabilities calculated at 3, 5, and 10 years in the KPNC cohort.

|  | **3 years** | **5 years** | **10 years** |
| --- | --- | --- | --- |
| CVD mortality | 0.84 (0.82, 0.86) | 0.85 (0.83, 0.86) | 0.82 (0.81, 0.84) |
| Breast cancer mortality | 0.82 (0.80, 0.84) | 0.80 (0.78, 0.87) | 0.77 (0.75, 0.79) |

Supplementary Table 5: Cardiovascular disease risk factors in the KPNC cohort compared to Framingham women

|  | KPNC | | Framingham |
| --- | --- | --- | --- |
|  | N | % | % |
| Age | 60.0 (mean) | (SD=12.8) | 49.6 (SD=11.1) |
| HDL Cholesterol |  |  |  |
| <35 | 7,338 | 35.9 | 4 |
| 35-44 | 4,957 | 24.2 | 15 |
| 45-49 | 2,342 | 11.5 | 12 |
| 50-59 | 2,928 | 14.3 | 28 |
| 60+ | 515 | 2.5 | 41 |
| Missing | 2,382 | 11.6 | 0 |
| Total Cholesterol |  |  |  |
| <160 | 2,350 | 11.5 | 8 |
| 160-199 | 6,431 | 31.4 | 30 |
| 200-239 | 6,191 | 30.3 | 33 |
| 240-279 | 2,571 | 12.6 | 20 |
| 280+ | 750 | 3.7 | 9 |
| missing | 2,169 | 10.6 | 0 |
| Systolic blood pressure |  |  |  |
| <120 | 5,145 | 25.1 | 35 |
| 120-129 | 4,967 | 24.3 | 21 |
| 130-139 | 4,600 | 22.5 | 15 |
| 140-149 | 2,179 | 10.7 | 19 |
| 150-159 | 1,499 | 7.3 |  |
| 160+ | 914 | 4.5 | 10 |
| missing | 1,158 | 5.7 | 0 |
| On blood pressure  lowering medication | 5,499 | 26.9 | n/a |
| Smoking status |  |  |  |
| No | 7,389 | 36.1 | 62 |
| Current | 2,430 | 11.9 | 38 |
| Quit | 3,095 | 15.1 | n/a |
| Missing | 7,548 | 36.9 | 0 |
| Diabetic | 2,669 | 13.0 | 4 |
| History of prior CVD event | 2,854 | 14.0 | 0 |

Supplementary Table 6: Parameter estimates and relative risks for Framingham model recalibrated to KPNC data set on complete cases (N=11,019)

|  | Framingham Women | | KPNC Breast Cancer Survivors | |
| --- | --- | --- | --- | --- |
|  | Beta | RR (95% CI) | Beta | RR (95% CI) |
| **Age** | 0.17 | 1.19 (0.97-1.45) | 0.04 | 1.04 (0.99, 1.10) |
| **Age squared** | 0.001 |  | 0.0002 |  |
| **Blood Pressure** |  |  |  |  |
| <120 | -0.74 | 0.48 (0.22-1.05) | -0.14 | 0.87 (0.72, 1.06) |
| 120-129 | reference |  | reference |  |
| 130-139 | −0.37 | 0.69 (0.34-1.42) | 0.06 | 1.06 (0.90, 1.26) |
| 140-159 | 0.22 | 1.24 (0.69-2.24) | -0.027 | 0.97 (0.81, 1.17) |
| 160+ | 0.61 | 1.84 (1.00-3.39) | 0.299 | 1.35 (1.01, 1.79) |
| **Total Cholesterol** |  |  |  |  |
| <160 | 0.21 | 1.23 (0.27-5.64) | -0.009 | 0.99 (0.82, 1.19) |
| 160-199 | reference |  | reference |  |
| 200-239 | 0.44 | 1.55 (0.81-2.96) | -0.177 | 0.84 (0.71, 0.99) |
| 240-279 | 0.56 | 1.74 (0.90-3.40) | 0.058 | 1.06 (0.86, 1.30) |
| 280+ | 0.89 | 2.44 (1.21-4.93) | -0.045 | 0.96 (0.68, 1.36) |
| **HDL Cholesterol** |  |  |  |  |
| <35 | 0.73 | 2.08 (1.00-4.31) | 0.513 | 1.67 (1.22,2.29) |
| 35-44 | 0.60 | 1.82 (1.05-3.16) | 0.252 | 1.29 (1.07,1.55) |
| 45-49 | 0.60 | 1.82 (1.05-3.14) | 0.121 | 1.13 (0.92,1.39) |
| 50-59 | reference |  | reference |  |
| 60+ | −0.54 | 0.58 (0.33-1.02) | -0.138 | 0.87 (0.74,1.03) |
| **Diabetes** | 0.87 | 2.38 (1.40-4.06) | 0.808 | 2.24 (1.94,2.60) |
| **Current smoker** | 0.98 | 2.65 (1.77-3.97) | 0.529 | 1.70 (1.46,1.98) |

Supplementary Table 7: Hazard Ratios from the Multi-state Model of Cause of Death Following a Breast Cancer Diagnosis in KPNC Women

|  | **CVD Mortality**  **696 events**  **HR (95%CI)** | **Breast Cancer Mortality**  **842 events**  **HR (95% CI)** | **All Other Causes**  **1,191 events**  **HR (95%CI)** |
| --- | --- | --- | --- |
| **Age categories** |  |  |  |
| 21-39 | Reference | Reference | Reference |
| 40-49 | 0.81 (0.13,1.49) | 1.09 (0.76, 1.42) | 0.63 (0.21, 1.05) |
| 50-54 | 1.03 (0.34,1.72) | 0.97 (0.62, 1.32) | 0.65 (0.21, 1.09) |
| 55-59 | 1.04 (0.36,1.72) | 1.11 (0.77, 1.45) | 1.02 (0.61, 1.43) |
| 60-64 | 1.34 (0.68,2.00) | 1.30 (0.96, 1.64) | 1.04 (0.63, 1.45) |
| 65-69 | 1.88 (1.23,2.53) | 1.19 (0.83, 1.55) | 1.44 (1.04, 1.84) |
| 70-74 | 3.40 (2.76,4.04) | 1.65 (1.29, 2.01) | 2.45 (2.06, 2.84) |
| 75+ | 6.27 (5.64,6.90) | 2.47 (2.15, 2.79) | 4.02 (3.63, 4.41) |
| **Race** |  |  |  |
| White | 1.42 (1.09,1.75 ) | 1.30 (1.06, 1.54) | 1.47 (1.23, 1.71) |
| Black | 2.53 (2.15,2.91) | 1.64 (1.33, 1.95) | 1.66 (1.36, 1.96) |
| Asian/Pacific Islander | Reference | Reference | Reference |
| Other/Unknown | 1.48 (0.06,2.90) | 1.65 (1.33, 1.95) | 1.41 (0.25, 2.57) |
| **Smoking Status** |  |  |  |
| Current | 2.59 (2.32,2.86) | 1.96 (1.71, 2.21) | 3.08 (2.87, 3.29) |
| Former | 1.36 (1.09,1.63) | 1.33 (1.08, 1.58) | 1.49 (1.28, 1.70) |
| Non-smoker | Reference | Reference | Reference |
| Unknown | 2.28 (2.07,2.49) | 2.29 (2.11, 2.47) | 2.69 (2.52, 2.86) |
| **History of CVD** |  |  |  |
| No prior history | Reference | - | Reference |
| History of event | 2.10 (1.89,2.31) | - | 1.28 (1.11, 1.45) |
| Unknown | 1.53 (1.19,1.87) | - | 1.44 (1.17, 1.71) |
| **HDL Cholesterol** |  |  |  |
| <35 | 1.54 (1.14,1.94 ) | 1.92 (1.59, 2.25) | 1.91 (1.62, 2.20) |
| 35-44 | 1.04 (0.79,1.29) | 1.28 (1.07, 1.49) | 1.14 (0.95, 1.33) |
| 45-49 | 0.84 (0.54,1.14) | 0.90 (0.65, 1.15) | 1.04 (0.83, 1.25) |
| 50-59 | 0.81 (0.58,1.04) | 0.99 (0.80, 1.18) | 0.97 (0.80, 1.14) |
| 60+ | Reference | Reference | Reference |
| Unknown | 1.32 (1.06, 1.58) | 1.57 (1.36, 1.78) | 1.49 (1.27, 1.71) |
| **Charlson comorbidity** |  |  |  |
| 0 | Reference | - | Reference |
| 1-2 | 1.44 (1.26,1.62) | - | 1.54 (1.41, 1.67) |
| 3+ | 2.28 (2.03,2.53) | - | 2.55 (2.34, 2.76) |
| **Grade** |  |  |  |
| Well differentiated | Reference | Reference | Reference |
| Moderately differentiated | 1.11 (0.90,1.32) | 2.86 (2.51, 3.21) | 1.18 (1.02, 1.34) |
| Poorly differentiated | 1.45 (1.20,1.70) | 4.34 (3.99, 4.69) | 1.56 (1.37, 1.75) |
| Diffuse | 1.05 (0.28,1.82) | 4.59 (4.03, 5.15) | 1.02 (0.43, 1.61) |
| Unknown | 1.52 (1.24, 1.80) | 2.63 (2.23, 3.03) | 1.12 (0.89, 1.35) |
| **Tumor size** |  |  |  |
| ≤2 cm | Reference | Reference | Reference |
| (2,5] cm | 1.43 (1.25,1.61) | 2.13 (1.96, 2.30) | 1.38 (1.24, 1.52) |
| >5 cm | 1.91 (1.57, 2.25) | 3.19 (2.95, 3.43) | 2.01 (1.75, 2.27) |
| Diffuse or Unknown | 3.74 (3.20,4.28) | 5.26 (4.92, 5.60) | 2.91 (2.43, 3.39) |
| **Lymph Nodes Involved** |  |  |  |
| 0 | Reference | Reference | Reference |
| 1-3 | 1.23 (1.03,1.43) | 1.71 (1.54, 1.88) | 1.13 (0.98, 1.28) |
| 4-9 | 1.51 (1.21,1.81) | 3.04 (2.83, 3.25) | 1.55 (1.32, 1.78) |
| 10+ | 2.35 (1.95,2.75) | 6.48 (6.25, 6.71) | 1.90 (1.58, 2.22) |
| Unknown | 2.52 (1.51,3.53) | 2.13 (0.72, 3.54) | - |
| **Radiation** | 0.82 (0.64,0.99) | - | 0.87 (0.73, 1.01) |
| **Chemotherapy** | 0.77 (0.55,0.99) | - | 0.83 (0.66, 1.00) |
| **Surgery** |  |  |  |
| None | 1.95 (1.59,2.31) | 3.25 (2.95, 3.55) | 1.98 (1.69, 2.27) |
| Lumpectomy | Reference | Reference | Reference |
| Mastectomy | 1.05 (0.88,1.22) | 1.33 (1.17, 1.49) | 1.07 (0.94, 1.20) |
| Unknown | 3.35 (1.93,4.77) | 1.96 (0.03, 3.95) | - |
| **ER/PR** |  |  |  |
| Positive | 0.82 (0.59,1.05) | 0.48 (0.31, 0.65) | 0.77 (0.60, 0.94) |
| Negative | Reference | Reference | Reference |
| Unknown/not done | 0.67 (0.41,0.93) | 0.53 (0.34, 0.72) | 0.63 (0.43, 0.83) |

Supplemental Table 8. Comparison of KPNC Cohort with SEER18.

|  |  |  |  |  |
| --- | --- | --- | --- | --- |
|  |  | **KPNC** | **SEER18** |  |
|  | **Age** (years) |  |  |  |
|  | < 35 | 1.5% | 1.9% |  |
|  | 35-39 | 2.8% | 3.3% |  |
|  | 40-44 | 6.6% | 7.1% |  |
|  | 45-49 | 10.6% | 10.8% |  |
|  | 50-54 | 12.7% | 12.0% |  |
|  | 55-59 | 14.5% | 12.5% |  |
|  | 60-64 | 14.0% | 12.1% |  |
|  | 65-69 | 12.1% | 10.9% |  |
|  | 70-74 | 9.8% | 9.6% |  |
|  | 75+ | 15.5% | 19.8% |  |
|  | **Race** |  |  |  |
|  | Non-Hispanic White | 69.3% | 74.1% |  |
|  | Non-Hispanic Black | 7.3% | 9.6% |  |
|  | Hispanic | 9.8% | 8.8% |  |
|  | Asian/Pacific Islander | 13.3% | 6.8% |  |
|  | Other/Unknown | 0.3% | 0.7% |  |
|  | **Stage** |  |  |  |
|  | 1 | 53.0% | 50.7% |  |
|  | 2 | 38.2% | 35.7% |  |
|  | 3 | 8.9% | 13.7% |  |
